# Pregnancy Complications Lead to Subclinical Maternal Heart Dysfunction—The Importance and Benefits of Follow-Up Using Speckle Tracking Echocardiography

**DOI:** 10.3390/medicina58020296

**Published:** 2022-02-15

**Authors:** Mihaela Roxana Popescu, Alexandra Bouariu, Anca Marina Ciobanu, Nicolae Gică, Anca Maria Panaitescu

**Affiliations:** 1 Cardiothoracic Pathology Department, “Carol Davila” University of Medicine and Pharmacy, 020021 Bucharest, Romania; 2 Department of Cardiology, Elias University Emergency Hospital, 011461 Bucharest, Romania; 3 Department of Obstetrics and Gynecology, Filantropia Clinical Hospital, 011171 Bucharest, Romania; alexandra.bouariu@yahoo.com (A.B.); ciobanu.ancamarina@gmail.com (A.M.C.); nicolae.gica@umfcd.ro (N.G.); anca.panaitescu@umfcd.ro (A.M.P.); 4 Department of Obstetrics and Gynecology, “Carol Davila” University of Medicine and Pharmacy, 020021 Bucharest, Romania

**Keywords:** preeclampsia, gestational diabetes, speckle-tracking, echocardiography, long-term cardiovascular disease, heart failure preserved ejection fraction

## Abstract

Pregnancy complications such as gestational diabetes (GDM) and hypertensive disorders of pregnancy (HDP) are frequent and influence not only fetal outcomes but also the maternal cardiac function. GDM and HDP may act as a proxy for increased metabolic and cardiovascular risk later in life. Speckle tracking echocardiography (STE) is a relatively new imaging technique that provides more sensitive assessment than conventional echocardiography of the maternal cardiac function. Recent research suggests that STE can be used during pregnancy and postpartum as a useful method of early detection of subclinical maternal cardiac changes related to pregnancy complications, such as GDM and HDP, and as an indicator for future maternal cardiovascular disorders. The aim of this review was to underline the current value of STE in the follow-up protocol of high-risk pregnant women, as a mean for pre- and postpartum monitoring. A review of the literature was conducted in the PubMed database to select relevant articles regarding the association of STE changes and HDP or GDM in the prenatal and postpartum maternal evaluations. Both GDM and HDP are associated with subtle myocardial changes in shape, size and function; these preclinical cardiac changes, often missed by conventional evaluation, can be detected using STE. Left ventricular global circumferential strain might be an important predictor of maternal cardiovascular disorders and might help to define a high-risk group that requires regular monitoring later in life and timely intervention.

## 1. Introduction

Echocardiography is a consecrated diagnostic and follow-up method for the adult population with cardiovascular (CV) risk factors and cardiac disease. In pregnancy, maternal echocardiography is reserved for selected cases, with previous cardiac pathologies or onset of symptoms suggesting heart failure either when assessed by the cardiologist or for research purposes, as observed in the recent literature [1,2] A new approach, by assessing the cardiac specific changes during pregnancy-related complications and even postpartum follow-up, can detect early changes that are indicative of future cardiovascular disease (CVD). Detection of subclinical myocardial dysfunction might be an indication for the need of closer postpartum follow-up, as retrospective studies have shown that a significant number of patients with heart failure with preserved ejection fraction have experienced a preeclamptic pregnancy [3].

The aim of this review is to underline the value of newer echocardiography techniques in the follow-up protocol of high-risk pregnancies, as a mean for pre- and post-partum monitoring. Pregnancy complications, such as gestational diabetes (GDM) or hypertensive disorders of pregnancy (HDP), can foreshadow subsequent cardiovascular and metabolic disease in future life [4]. Therefore, maternal fetal specialists and cardiologists as a team need to be aware that women suffering from these complications should undergo regular postpartum monitoring. Echocardiography, and in particular, speckle tracking echocardiography, could prove to be a clinically useful tool for this joint effort for a better follow-up.

## 2. General Methods of Assessing Cardiac Function; Speckle Tracking Echocardiography as a Potential Candidate for Maternal Cardiac Evaluation in Pregnancy Complications and Thereafter

Specific and standardized assessment of the cardiac function has been the subject of broad and permanent interest [5,6]. The assessment of the cardiac function aimed to provide important diagnostic and prognostic information with regards to normal heart function or postinfarction recovery, left ventricular hypertrophy or chronic heart failure [7]. Clinical assessment of the cardiac function provides informative evaluation of the heart function but is generally poor [8]. Electrocardiogram is a non-invasive investigation technique that possesses the disadvantage of nonspecific findings, although an entirely normal electrocardiogram has a 95% likelihood of normal systolic function [9]. Non-invasive cardiac output monitoring, (NICOM), a new method to measure cardiac output based on bioreactance, which records the relative phase shifts of oscillating alternating currents across the thorax, calculates several hemodynamic measurements and has been demonstrated to be similarly consistent with ultrasound assessment [10]. Its potential in monitoring pregnancy complications is yet to be demonstrated. Left ventricular function has been reported to be a powerful predictor of long-term survival in patients affected by a broad spectrum of cardiac diseases. The most widely used echocardiographic parameter to quantify LV systolic function has been LV ejection fraction (LVEF). While LVEF is a strong predictor of mortality, it is highly load-dependent, depends critically on operator expertise and is affected by significant interobserver and interobserver variability [11]. Assessments such as computed tomography and radionuclide ventriculography (either Tc-99m-labeled red blood cells or human serum albumin), while useful in the general population, subject the patient to unnecessary radiation [12,13]. Cardiovascular magnetic resonance is also useful for cardiac function assessment [14], but is time consuming and costly.

However, echocardiography is a widely available, non-invasive, not irradiating technique, suitable for maternal heart monitoring in pregnancy and postpartum. Its main disadvantages are related to image acquisition, which is operator- and acoustic-window dependent and to that the assessment of ventricular function is related to geometric assumptions of the normal heart, with less reliability in some cases [15].

**Figure 1:**
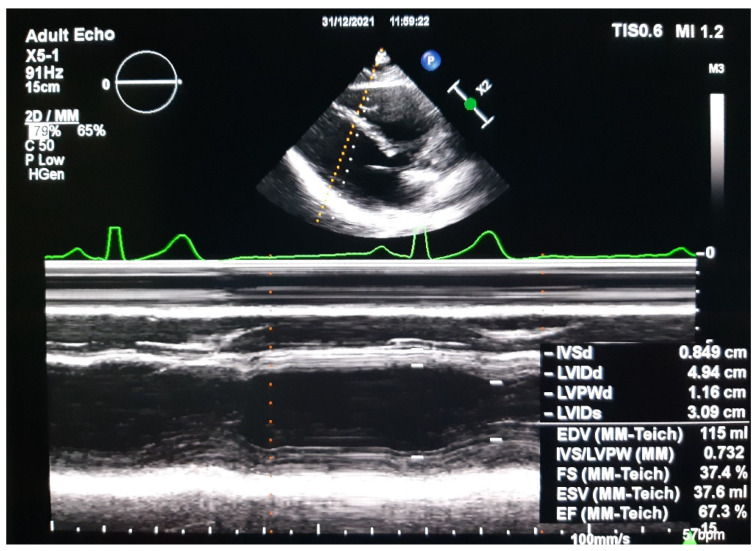
M-Mode echocardiography of a healthy pregnant patient, in parasternal long-axis view showing left ventricular fractional shortening and ejection fraction–IVSd (interventricular septum thickness at diastole, LVIDd (left ventricle internal diameter during diastole), LVIDs (left ventricular internal diameter during systole, LVPWd (left ventricular posterior wall thickness during diastole, EDV (end diastole volume), ESV (end systole volume), EF (ejection fraction), FS (fractional shortening). Courtesy of Elias Cardiology Department.

M-Mode echocardiography, a method that measures the mid-left ventricle diameters in the short axis and volumes obtained by cubing the diameters, estimates left ventricular fractional shortening and ejection fraction [16] (Figure 1). A more accurate measurement than M-mode is area-length assessment in two-dimensional echocardiography. This method possesses a moderate reproducibility, as it relies on good endocardial border definition that is highly subjective but can be clinically demonstrated with experience [17].

**Figure 2:**
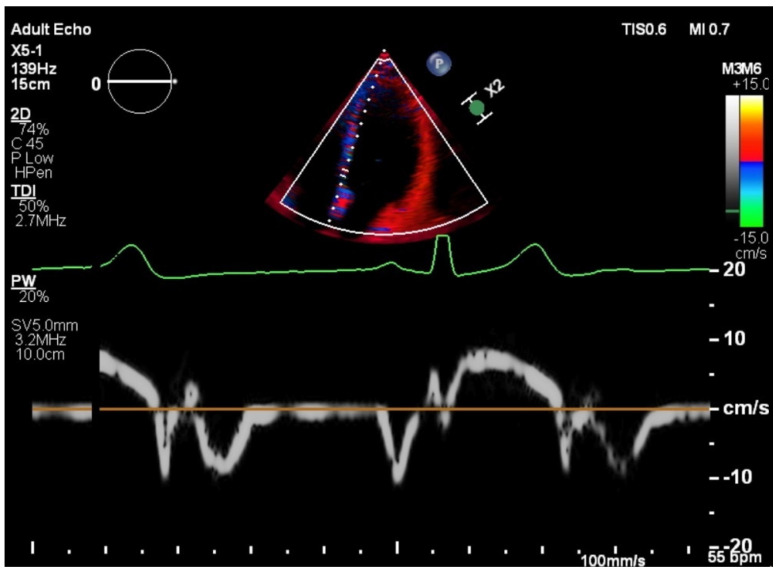
Tissue Doppler imaging echocardiography of a healthy pregnant patient in apical 4 chamber view, septal interogation–MV e’ Vel lat (mitral valve e wave velocity), MV a’ Vel lat (mitral valve a wave velocity), MV s’ Vel lat (mitral valve s wave velocity), E/E’ lat = relaxation ratio (E peak early mitral valve inflow velocity, E’ early mitral valve diastolic velocity). Courtesy of Elias Cardiology Department.

Global myocardial performance has been assessed by other echocardiographic methods, derived from Doppler analysis, such as myocardial performance index (Tei index) and tissue Doppler mitral annular systolic velocity (Figure 2). These methods do not require endocardial border definition and do not rely on subjective geometric assumption but require a good alignment of the ultrasound beam with blood flow and with myocardial motion [18].

Another approach to assess left cardiac function is three-dimensional echocardiography that eliminates the geometric assumptions and operator dependent measurements [19]. 

A relatively new tool for assessing left ventricular function through myocardial strain with a high temporal and spatial resolution is speckle-tracking imaging [20]. This method possesses better inter- and intra-observer variations. It can assess, simultaneously, the entire myocardium along the 3D geometrical (longitudinal, circumferential and radial) axis and is not affected by translation cardiac movements; it is angle dependent and possesses a low spatial and temporal resolution [20].

Currently, speckle tracking echocardiography is the most applicable method in the context of pregnancy related complications and has shown the most promising results in detection of subclinical myocardial dysfunction. Two-dimensional speckle-tracking echocardiography is proposed as a novel technique for objective and quantitative evaluation of global and regional myocardial function. The myocardial deformation strains are obtained by frame-by-frame automatic measurement of the distance between two points of each LV segment during the cardiac cycle. The ST allows quantification of myocardial or more correct endocardial deformation. This technique enables us to calculate the segmental, as well as the global longitudinal, strain and strain rate. It measures myocardial velocities and deformation parameters and has evolved to be the imaging modality of choice to detect subclinical cardiac dysfunction [21].

## 3. Clinical Applications of Speckle Tracking Echocardiography

Subclinical cardiac dysfunction precedes the development of heart failure and other cardiovascular diseases, but often goes undiagnosed by conventional echocardiography. Recognition of subclinical myocardial dysfunction offers clinicians an opportunity for early intervention and prevention of symptomatic cardiovascular disease and novel imaging method, such as STE, might be a useful screening strategy. For example, subclinical LV systolic dysfunction in the general population with diabetes mellitus, detected using LV global longitudinal strain, is associated with an increased incidence of cardiovascular events, defined as a composite of acute coronary syndrome, cerebrovascular stroke, cardiovascular death and hospitalization for heart failure [22,23].

Speckle tracking echocardiography is an advanced, non-invasive imaging technique that provides fast and accurate assessment of cardiac function, global and regional function of both atrial and ventricular chambers, in-plane translation motion and independently from the angle of insonation [24]. The information obtained regarding the cardiac deformation is similar to the assessment of cardiovascular magnetic resonance [21].

It has been demonstrated over the last 10 years that speckle tracking echocardiography is a reliable method in predicting heart failure. Studies in non-pregnant population showed that there is a specific pattern of changes when using speckle tracking, not only in arterial hypertension, but also following diabetes mellitus [25]. Early signs of heart function deterioration can be detected in coronary artery disease and valvular disorders by using speckle tracking imaging [25,26].

**Figure 3:**
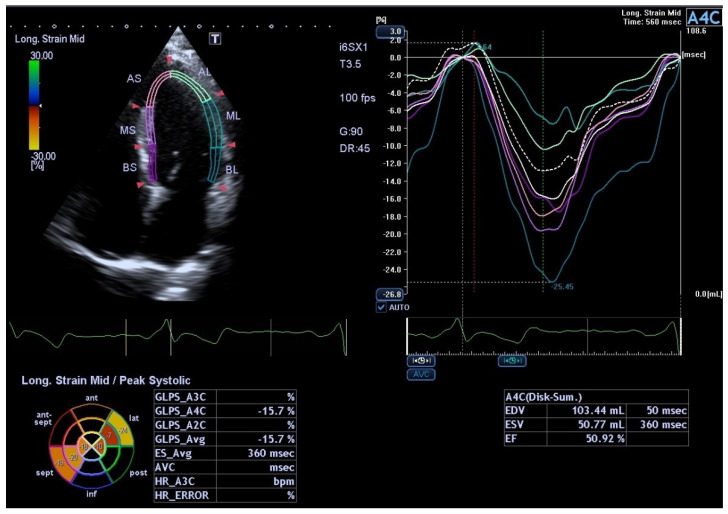
Left ventricular function analysis in a pregnant patient, 4 chamber apical view. AS—apical septal, AL—apical lateral, ML—mid lateral, MS—mid septal, BL—basal lateral, BS—basal septal, EDV—end-diastolic volume, ESV—end-systolic volume, EF—ejection fraction.

In comparison with other methods of assessing heart function, the main benefit of SPE is represented by the capacity to evaluate the two-dimensional displacement of cardiac muscle spots during the cardiac cycle and to calculate, in an angle-independent way, heart deformation through the distance variation between the speckles in the analysed segment (Figure 3). Therefore, reconstruction of the spatial deformation of the left ventricular heart in different spatial plane (longitudinal, radial, circumferential and rotational) facilitates an accurate cardiac function less dependent on operator variability of the analysis compared to standard echocardiography [24].

Myocardial deformation during cardiac cycle, contraction and relaxation, is defined by the term strain [25]. It represents the grade of the deformation of an analysed segment and is expressed as a percentage (%). In the assessment of longitudinal function, negative values of strain indicate shortening or compression of the object, while positive values testify myocardial stretching and expansion. On the other hand, the strain rate is the frequency per time unit at which the deformation occurs [24].

**Figure 4:**
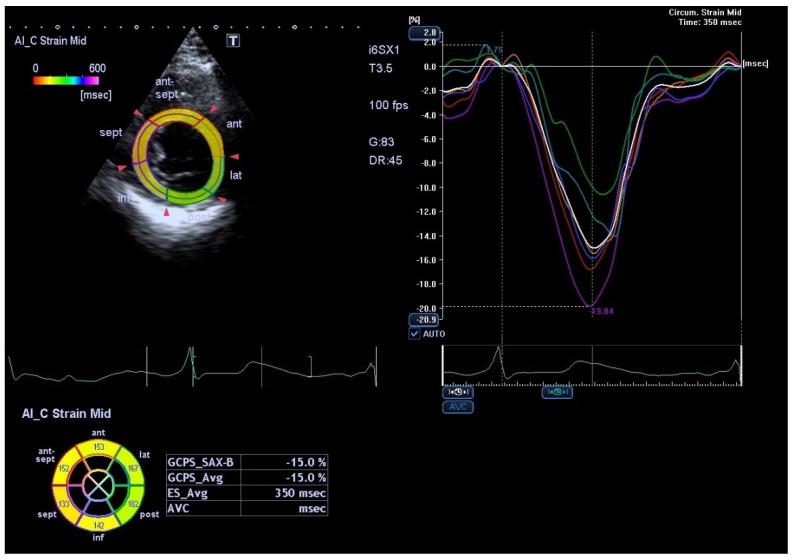
Echocardiography of a healthy pregnant patient showing circumferential left ventricle strain analysis (at the level of the papillary muscles) and speckle tracking analysis. GCPS_SAX-M-Global circumferential strain peak systolic middle left ventricle; GCPS_Avg-Average global circumferential strain; ES_Avg-Average End systolic time. Courtesy of the Fetal Medicine Foundation.

The adult myocardium possesses a complex structure, based on right-handed helical fibers at endocardial layer and left-handed helical fibers in epicardial layer [5]. Harmonic contraction of all these fibers allows cardiac deformation in different space planes, such as longitudinal (systolic shortening and diastolic stretching), radial (systolic thickening) and circumferential (shortening during systole and stretching during diastole) planes (Figure 4) [24]. Moreover, during systole, a counterclockwise rotation of the apex and a clockwise rotation of the base occur with consequent opposite rotations during the following diastole. These movements are simultaneous and determine the left ventricular systolic twisting or torsion and diastolic untwisting [2]. Longitudinal strain is described as myocardial shortening along its longitudinal axis and represents a negative curve during systole and a positive one during diastole and can be evaluated in apical 2-, 3- and 4-chamber views [2]. Longitudinal strain offers an accurate early detection of the alterations that may affect the subendocardial longitudinal fibers, such as an ischemic injury [26] or arterial hypertension [27]. These findings may be extremely useful in a subclinical phase when left ventricular ejection fraction is typically normal.

Defined by a positive curve, radial strain shows the radial myocardial deformation corresponding to systolic thickening. This parameter is assessing left ventricular dyssynchrony and, hence, has proved to be one of the most accurate methods to identify potential responders to cardiac resynchronization therapy in patients with end-stage heart failure [20]. Circumferential fibers are placed at mid-wall and participate, equally to and together with longitudinal and radial ones, to maintain heart function within normal limits [28].

The rotation degrees of the intermediate myocardial planes gradually change from base to apex and the rotation is null at papillary muscles level. A parasternal short-axis section at basal and apical planes can assess left ventricular twisting value [28].

## 4. Results: Speckle Tracking Echocardiography in Pregnancy Complications

### 4.1. Gestational Diabetes Mellitus

Gestational diabetes affects one out of seven pregnancies and is associated with adverse perinatal outcomes for both the mother and the fetus. Women whose pregnancies were complicated with GDM have a seven-fold higher incidence of type 2 diabetes later in life and a two-fold higher risk of cardiovascular events during the first 10 years postpartum [29] Moreover, gestational diabetes mellitus changes the fetal balanced environment, influencing fetal cardiac development and predisposing to cardiovascular disease in children and young adults [30]. The same environment that shows insulin resistance influences the maternal heart, demonstrating mild and transient findings that may be a predictor of cardiovascular disease later in life [31].

We did a search on PubMed with no time restriction for words such as “echocardiography” and “pregnancy” and “gestational diabetes”. We retrieved 354 results out of which only 19 were related to maternal cardiac echocardiography in relation to diabetes. After reading the studies, we found eight studies that used STE and reported subtle differences between cardiac function in pregnant women with and without diabetes. (Table 1).

Increased post-partum cardiovascular risk after GDM might be explained by subclinical cardiac changes which appear during pregnancy in women exposed to high glycemic levels and insulin resistance. Most of the studies reported early changes at STE; the main finding when comparing the pregnancies complicated by GDM to normal population is reduced left ventricular global longitudinal strain (LV-GLS).

A recent large prospective longitudinal study [31] enrolled 161 third-trimester women diagnosed with gestational diabetes defined according to NICE guidelines [48] and 438 women with uncomplicated pregnancies and compared maternal and fetal heart changes in pregnancy using speckle tracking echocardiography assessment. Women with GDM showed higher left ventricular mass, lower tissue Doppler systolic wave and lower global longitudinal strain. The left atrial area was higher than the control group and there was prolonged isovolumic relaxation time. There were no differences between the groups in terms of cardiac output and peripheral vascular resistance [31].

Gestational diabetes also has an impact on the offspring cardiac function, manifesting early signs of subclinical cardiac dysfunction since intrauterine life. A recent study illustrated that fetuses of mothers with gestational diabetes mellitus possessed more globular hearts with higher right and left ventricular sphericity index (*p* < 0.001 for both) and lower left ventricular ejection fraction. Early subclinical systolic functional impairment is easily proven by speckle tracking in fetuses with gestational diabetic mothers; functional changes were more evident in the right ventricle [31]. Similar findings, including changes of fetal heart shape and biventricular diastolic disfunction at speckle tracking analysis were shown in two other studies [49,50]. Despite maternal diabetic treatment, neonatal follow-up at 6–7 months of age, showed persistent heart remodeling and impairment in diastolic and systolic ventricular function, with lower left ventricular global longitudinal systolic strain [51]. Similar to neonatal persistent changes, maternal subclinical heart dysfunction persists to at least 6 months alter delivery [33,36,37].

When comparing with other literature data, the results are poor; only a few studies assessed fetal heart in gestational diabetes mothers in the neonatal period. Similar results, with persistent alterations in left ventricular chamber geometry, were seen in the study of Patey et al. [49]. Decreased left ventricular systolic and diastolic function in the first week of life was shown in Zablah et al.’s report [52]. The only study that reported on transient fetal cardiac that spontaneously resolved after birth is the study of Mehta et al. [53]. It must be considered that the postpartum period is characterized by changes in fetal circulation and physiological adaptation to postnatal life [31]. This may influence the differences between fetal and neonatal life; therefore, further studies are needed to establish the changes associated with gestational diabetes.

Regarding the additive value of STE performed during pregnancy in prediction of GDM, the data show that, although subtle hemodynamic and functional cardiac changes appear prior to the development of gestational diabetes, maternal cardiac evaluation does not offer additional predictive information in pregnancy outcomes compared to demographic characteristics and medical history [42].

### 4.2. Speckle Tracking Echocardiography in Preeclampsia, Chronic Hypertensive and Fetal Growth Restriction

Hypertensive disorders affect around 15% of all pregnancies and account for 16% of all maternal deaths worldwide, with an estimated 62,000 to 77,000 deaths per year [54]. There is a significant risk of cardiovascular morbidity associated to HDP; around 20% of women with preeclampsia remain hypertensive at 6 months postpartum; these women are at a 3-fold increased risk of chronic hypertension [55,56]. Moreover, a history of preeclampsia is associated with a 2- to 4-fold increased risk of heart failure, coronary artery disease, stroke, and cardiovascular disease–related death [57]. The American Heart Association has now recognized both gestational hypertension and preeclampsia as risk factors for cardiovascular disease [58]. Therefore, adequate screening methods for early prediction of preclinical stage of cardiac dysfunction and long-term postpartum follow-up are essential for reducing maternal morbidity and mortality from cardiovascular complications [59,60].

Over the last few years, STE and global longitudinal strain (GLS) was extensively studied in the context of hypertensive disorders of pregnancy. Left ventricular-GLS represents LV myocardial shortening in the longitudinal axis and is an important index of global LV function. It is an early marker of subclinical alterations of subendocardial longitudinal fibers, which are the first to be affected in ischemic injuries and arterial hypertension [25]. Although left ventricular ejection fraction is the most widely used parameter in current clinical practice for assessing cardiac function, it holds several limitations, mainly late decrease only in the advanced stage of heart disease. The latest data have reported that LV global longitudinal strain is a more sensitive measure of preclinical myocardial dysfunction and is more reproducible than left ventricular ejection function evaluated through conventional echocardiography [61].

A recent systematic review regarding STE findings in pregnancies complicated by hypertensive disorders analyzed 16 relevant studies and reported that LV-GLS was decreased in women with any form of hypertensive complication. Additionally, women with early onset or severe form of preeclampsia showed other deformation changes, such as decreased left ventricular global radial and circumferential strain [62]. These preclinical changes in myocardial function last for almost 10 years after delivery; STE might be used as a sensitive method for early detection of women at high risk who can benefit from regular cardiovascular monitoring [63].

Reduced sensitivity of conventional echocardiography in detecting subclinical cardiac changes is supported by the results of a systematic review on women with a history of preeclampsia assessed by conventional echocardiography, which failed to show any difference in left ventricular ejection fraction, isovolumetric relaxation time, or deceleration time. The results suggest that cardiac morbidity associated with preeclampsia is a result of an increased incidence of the cardiovascular risk factors within the population group (metabolic syndrome, renal disease, diabetes mellitus), rather than the preeclampsia alone. In terms of measurement of global myocardial deformation, speckle tracking imaging is a useful tool to assess maternal cardiac function, showing reduced strain in preeclampsia as compared to normotensive pregnancies [64].

A prospective observational study [65] including women attending for a routine second trimester ultrasound showed speckle tracking evidence of altered cardiac geometry, increased in peripheral vascular resistance and impaired myocardial function in pregnancies that subsequently developed preeclampsia. Peripheral vascular resistance was the cardiovascular parameter that was significantly affected by subsequent preeclampsia after adjustment for maternal demographic characteristics and medical history [65]. However, maternal cardiovascular parameters are not useful in the prediction of preeclampsia but provide information on the pathophysiology of preeclampsia.

Another study published in 2020 by Vasapollo et al. [66] reported cardiac changes in pregnant women known with chronic hypertension on medication and pre-pregnancy left ventricular dysfunction and remodeling. They assessed maternal heart comparing early and late complications, such us superimposed preeclampsia, fetal growth restriction and HELLP. Using speckle tracking echocardiography, the group reported that left ventricle hypertrophy and concentric geometry is the strongest independent predictor of complications in the subsequent pregnancy. Concentric geometry appears in early complications, whereas eccentric hypertrophy appears in late complications [65]. With regards to diastolic function, it seems to be an independent predictor of complications [65,66].

Assessing both preeclampsia and fetal growth restriction in fetal cardiac remodeling, Youssef et al. [67] demonstrated hypertrophic and globular hearts with increased myocardial performance index.

## 5. Discussions

During pregnancies affected by preeclampsia, mild diastolic dysfunction can be detected with standard echocardiographic techniques, but STE can also identify systolic dysfunction [68]. A recent report found that blood pressure variability affects the right ventricle as well [69,70]. Recent data shows that, compared to uncomplicated pregnancies, the ones complicated by preeclampsia affect cardiac function in the long-term, at 10 years post-partum [71]. Moreover, women with early onset preeclampsia showed reduced long-term left ventricular global longitudinal strain associated with a low coronary flow velocity reserve, as a sign of microvascular dysfunction [63] and of higher left ventricular mass [72]. Moreover, at 12 years follow-up, women with early onset preeclampsia demonstrated worse subclinical left ventricle dysfunction than the ones with late onset preeclampsia [73,74]. This is also confirmed by the fact that all three myocardial layers were more affected in early vs. late-onset preeclampsia in a study performed using layer specific STE [75]. Moreover, a very recent study demonstrated that preeclampsia can predict the risk of hospitalization for heart failure with preserved ejection fraction [76]. It is known that women represent the majority of patients with heart failure with preserved ejection fraction [77]. However, it is surprising and disquieting that the median time to heart failure onset was only 32.2 months postpartum in the previously mentioned study [76].

Furthermore, the subtle systolic and diastolic dysfunction observed in late pregnancy, when complicated by gestational diabetes, also persist for at least six months post-partum [36]. Long-term studies are warrantied for this at-risk group, in order to establish the value of the method and create an appropriate follow-up protocol [36]. Closely monitored high-risk pregnancies [44,78,79,80,81] will also lead to fewer fetal complications.

All these data support the coordinated use of speckle tracking by teams of maternal fetal specialists and cardiologists in patients with pregnancy complications, both pre and postpartum. The desired outcome would be to design tailored monitoring protocols for every clinical situation, in order to foresee possible long-term effects temporarily unmasked during pregnancy [81].

## 6. Conclusions

It may well be that speckle tracking is for hypertensive diseases of pregnancy what the oral glucose tolerance test is for gestational diabetes mellitus, both for pregnancy and for the long-term follow-up. The current literature is limited concerning the use of speckle tracking in patients with pregnancy complications such as maternal gestational diabetes, preeclampsia and fetal growth restriction and comparative studies. Focus on a larger number of cases with a continuous postnatal monitoring is needed, especially for complications such as preeclampsia, in order to improve early prediction of frequent maternal complications.

## Figures and Tables

**Table 1:** Studies assessing maternal cardiac function in gestational diabetes. DM 1-diabetes mellitus type 1, GDM-gestational diabetes mellitus, LV-left ventricle, STE-speckle tracking echocardiography. In bold, significant STE changes.

Author, Year	Study Design	Population	Main Findings in Relation to STE
Buddeberg BS et al. (2020) [32]	prospective case-control study; at term (>37 weeks)	GDM = 40 Control = 40	**In GDM STE shows significant reduction in LV global longitudinal strain, LV** **endocardial and epicardial global longitudinal strain**
Schnettler WT et al. (2021) [33]	retrospective cohort analysis; peri- and postpartum	GDM = 205	No STE findings associated with GDM
Company Calabuig AM et al. (2021) [34]	cross-sectional study; 26–40 weeks	GDM = 123 Control = 246	**In GDM STE shows lower left and right global longitudinal strain**
Meera SJ et al. (2017) [35]	retrospective cohort study	GDM = 18 Control = 72	**In GDM STE shows lower global longitudinal strain, greater time-to-peak strain**
Aguilera J et al. (2020) [36]	case-control study; 35–36 weeks’	GDM = 73 Control = 73	**In GDM STE shows lower global longitudinal systolic strain at 35–36 weeks and at 6 months postpartum**
Sonaglioni A et al. (2021) [37]	case-control study	GDM = 30 Control = 30	**In GDM STE shows increased LV mass index and lower relaxation ratio**
Ando T et al. (2015) [4]	retrospective cohort study; 2nd and 3rd trimester	Pregnancy complications (PE,GDM) = 74 Control = 21	No STE changes specific associated to GDM
Airaksinen KE et al. (1986) [38]	case-control study	DM1 = 17 Control = 11	No GDM changes smaller LV in DM1
Aguilera J et al. (2020) [31]	cross-sectional study;third trimester	GDM = 161 Control = 483	**In GDM lower left ventricular diastolic and systolic functional indices**
Appiah D et al. (2016) [39]	retrospective cohort study	GDM = 64	In GDM increased LV mass and impaired LV relaxation and systolic function
Lindley KJ et al. (2020) [40]	retrospective case-control study	Pregnancy complications (PE) = 86	No STE changes specific associated to GDM
Oliveira AP et al. (2015) [41]	case-control study	GDM = 21 Control = 23	**In GDM STE shows mild diastolic dysfunction**
Gibbone E et al. (2021) [42]	prospective observational study; 19–23 weeks’	GDM = 199 Control = 2654	**In GDM significant differences in E/A ratio, E/e’ ratio, myocardial performance index and global longitudinal systolic strain (through STE)**
Schannwell CM et al. (2003) [43]	case-control study	DM1 = 51 Control = 51	No GDM; delayed relaxation at the beginning of pregnancy and developed a restrictive filling pattern in DM 1
Freire CM et al. (2006) [44]	case-control study	GDM = 13 Control = 13	In GDM mild degree of diastolic abnormality
Ye X et al. (2021) [45]	prospective observational study	GDM = 57	In GDM expansion of the LA volume, a mirror of LV systolic function
Zakovicova E et al. (2014) [46]	observational study	GDM = 31 Control = 31	In GDM increased LV walls thicknesses
Pintaudi B et al. (2013) [47]	case-control study	GDM = 55 Control = 51	In GDM deteriorating cardiac diastolic function

Bold indicates significant STE changes.

## Data Availability

All images courtesy of Fetal Medicine Foundation and Elias Hospital Cardiology Department.

## References

[1] Sengupta S., Bansal S., Hofstra L., Sengupta P.P., Narula J. (2017). Gestational changes in left ventricular myocardial contractile function new insights from two-dimensional speckle tracking echocardiography. Int. J. Cardiovasc. Imaging.

[2] Onishi T., Saha S., Delgado-Montero A., Ludwig D.R., Onishi T., Schelbert E.B., Schwartzman D., Gorcsan J. (2015). Global longitudinal strain and global circumferential strain by speckle tracking echocardiography and feature-tracking cardiac magnetic resonance imaging: Comparison with left ventricular ejection fraction. J. Am. Soc. Echocardiogr..

[3] Beale A.L., Meyer P., Marwick T.H., Lam C.S.P., Kaye D.M. (2018). Sex differences in cardiovascular pathophysiology why women are overrepresented in heart failure withe preserved ejection fraction. Circulation.

[4] Panaitescu A.M., Roberge S., Nicolaides K.H. (2019). Chronic hypertension: Effect of blood pressure control on pregnancy outcome. J. Matern. Fet. Neonatl. Med..

[5] Ando T., Kaur R., Holmes A.A., Brusati A., Fujikura K., Taub C.C. (2015). Physiological adaptation of the left ventricle during the second and third trimesters of a healthy pregnancy: A speckle tracking echocardiography study. Am. J. Cardiovasc. Dis..

[6] Maceira M., Bellenger N.G., Pennell D.J. (2019). Assessment of cardiac function. Cardiovascular Magnetic Resonance.

[7] Rahimi K., Bennett D., Conrad N., Williams T.M., Basu J., Dwight J., Woodward M., Patel A., McMurray J., MacMahon S. (2014). Risk prediction in patients with heart failure: A systematic review and analysis. JACC Heart Fail..

[8] Marantz P.R., Tobin J.N., Wassertheil-Smoller S., Steingart R.M., Wexler J.P., Budner N., Lense L., Wachspress J. (1988). The relationship between left ventricular systolic function and congestive heart failure diagnosed by clinical criteria. Circulation.

[9] O’Keefe J., Zinsmeister A., Gibbons R. (1989). Value of normal electrocardiographic findings in predicting resting left ventricular function in patients with chest pain and suspected coronary artery disease. Am. J. Med..

[10] Squara P., Denjean D., Estagnasie P., Brusset A., Claude Dib J., Dubois C. (2007). Noninvasive cardiac output monitoring (NICOM): A clinical validation. Intensive Care Med..

[11] Mondillo S., Galderisis M., Mele D., Cameli M., Lomoriello V.S., Zaca V., Ballo P., D’Andrea A., Muraru D., Losi M. (2011). Speckle tracking echocardiography: A new technique for assessing myocardial function. J. Ultrasound Med..

[12] Hendel R., Berman D., Di Carli M., Heidenreich P.A., Henkin R.H., Pellikka P.A., Pohost G.M., Williams K.A. (2009). 2009 appropriate use criteria for cardiac radionuclide imaging. Am. Coll. Cardiol..

[13] Mahnken D., Heuzler E., Klotz E., Hannemuth A., Wildberger J.E., Gunther R.W. (2004). Determination of cardiac output with multislice spiral computed tomography: A validation study. Investig. Radiol..

[14] Bellenger N., Burgess M., Ray S., Lahiri A., Coats A.J., Cleland J.G., Pennell D.J. (2000). Comparison of left ventricular ejection fraction and volumes in heart failure by echocardiography, radionuclide ventriculography and cardiovascular magnetic resonance: Are they interchangeable?. Eur. Heart J..

[15] Teichholz L.E., Kreulen T., Herman M.V., Gorlin R. (1976). Problems in echocardiographic volume determinations: Echocardiographic-angiographic correlations in the presence or absence of asynergy. Am. J. Cardiol..

[16] Kronik G., Slany J., Mosslacher H. (1979). Comparative value of eight M-mode echographic formulas for determining left ventricular stroke volume. Circulation.

[17] Bellenger N.G., Francis J.M., Davies C.L., Coats A.J., Pennell D.J. (2000). Establishment and performance of a magnetic resonance cardiac function clinic. J. Cardiovasc. Magn. Reson..

[18] Carluccio E., Biagioli P., Alunni G., Murrone A., Zuchi C., Biscottini E., Lauciello R., Pantano P., Gentile F., Nishimura R.A. (2012). Improvement of myocardial performance (Tei) index closely reflects intrinsic improvement of cardiac function: Assessment in revascularized hibernating myocardium. Echocardiography.

[19] Hoffmann R., Barletta G., von Bardeleben S., Vanoverschelde J.L., Kasprazak J., Greis C., Becher H. (2014). Analysis of left ventricular volumes and function: A multicenter comparison of cardiac magnetic resonance imaging, cine ventriculography and unenhanced and contract enhanced two-dimensional and three-dimentional echocardiography. J. Am. Soc. Echocardiogr..

[20] Voigt J., Pedrizzetti G., Lysyansky P., Marwick T.H., Houle H., Baumann R., Pedri S., Ito Y., Abe Y., Metz S. (2015). Definitions for a common standard for 2D speckle tracking echocardiography: Consensus document of EACVI/ASE/Industry Task Force to standardize deformation imaging. Eur Heart J Cardiovasc. Imaging..

[21] Delgado V., Ypenburg C., van Bommel R., Tops L.F., Mollema S.A., Marsan N.A., Bleeker G.B., Schalij M.J., Bax J.J. (2008). Assessment of left ventricular dyssynchrony by speckle tracking strain imaging comparison between longitudinal, circumferential and radial strain in cardiac resynchronization therapy. J. Am. Coll. Cardiol..

[22] Holland D., Marwick T., Haluska B.A., Leano R., Hordern M.D., Hare J.L., Fang Z.H., Prins J.B., Stanton T. (2015). Subclinical LV dysfunction and 10-year outcomes in type 2 diabetes mellitus. Heart.

[23] Liu J., Chen Y., Yuen M., Zhen Z., Chan Z.W.S., Siu-Ling Lam K., Tse H.F., Yiu K.H. (2016). Incremental prognostic value of global longitudinal strain in patients with type 2 diabetes mellitus. Cardiovasc. Diabetol..

[24] Blessberger H., Binder T. (2010). Non-invasive imaging:two dimensional speckle tracking echocardiography: Basic principles. Heart.

[25] Cameli M., Mandoli G.E., Sciaccaluga C., Mondillo S. (2019). More than 10 years of speckle tracking echocardiography: Still a novel technique or a definite tool for clinical practice?. Echocardiography.

[26] Dumitriu-Leen A., Scholte A.J.H.A., Katsanos S., Hoogslag G.E., van Rosendael A.R., van Zwet E.W., Bax J.J., Delgado V. (2017). Influence of myocardial ischemia extent on left ventricular global longitudinal strain in patients after ST-segment elevation mycardial infarction. Am. J. Cardiol..

[27] Narayanan A., Aurigemma G.P., Chinali M., Hill J.C., Meyer T.E., Tighe D.A. (2009). Cardiac mechanics in mild hypertensive heart disease: A speckle-strain imaging study. Circ. Cardiovasc. Imaging.

[28] Sengupta P.P., Tajik A.J., Chandrasekaran K., Khandheria B.K. (2008). Twist mechanics of the left ventricle: Principles and application. JACC Cardiovasc. Imaging.

[29] Bellamy L., Casas J., Hingorani A.D., Williams D. (2009). Type 2 diabetes mellitus after gestational diabetes: A systematic review and meta-analysis. Lancet.

[30] Lee H., Jang H., Park H.K., Cho N.H. (2007). Early manifestations of cardiovascular disease risk factors in offspring of mothers with previous history of gestational diabetes mellitus. Diabetes Res. Clin. Pract..

[31] Aguilera J., Semmler J., Coronel C., Georgiopoulos G., Simpson J., Nicolaides K.H., Charakida M. (2020). Paired maternal and fetal cardiac functional measurements in women with gestational diabetes mellitus at 35–36 weeks’ gestation. Am. J. Obstet. Gynecol..

[32] Buddeberg B.S., Sharma R., O’Driscoll J.M., Kaelin Agten A., Khalil A., Thilaganathan B. (2019). The impact of gestational diabetes on maternal cardiac adaptation in pregnancy. Ultrasound Obstet. Gynecol..

[33] Schnettler W., Zinn C., Davaiah C.G., Wilson J. (2021). Indications for maternal echocardiography in detecting disease and the impact on pregnancy management. Am. J. Perinatol..

[34] Company Calabuig A.M., Nunez E., Sanchez A., Nicolaides K.H., Charakida M., De Paco Matallana C. (2021). Three-dimensional echocardiography and cardiac strain imaging in women with gestational diabetes mellitus. Ultrasound Obstet. Gynecol..

[35] Meera S.J., Ando T., Pu D., Manjappa S., Taub C.C. (2017). Dynamic left ventricular changes in patients with gestational diabetes: A speckle tracking echocardiography study. J. Clin. Ultrasound.

[36] Aguilera J., Sanchez Sierra A., Abdel Azim S., Georgiopoulos G., Nicolaides K.H., Charakida M. (2020). Maternal cardiac function in diabetes mellitus at 35–36 weeks’ gestation and 6 months postpartum. Ultrasound Obstet. Gynecol..

[37] Sonaglioni A., Barlocci E., Adda E., Esposito V., Ferrulli A., Nicolosi G.L., Bianchi S., Lombardo M., Luzi L. (2021). The impact of short-term hyperglycemia and obesity on biventricular and biatrial myocardial function assessed by speckle tracking echocardiography in a population of women with gestational diabetes mellitus. Nutr. Metab. Cardiovasc. Dis..

[38] Airaksinen K., Ikaheimo M., Salmela P.I., Kirkinen P., Linnaluoto M.K., Takkunen J.T. (1986). Impaired cardiac adjustment to pregnancy in type I diabetes. Diabetes Care.

[39] Appiah D., Schreiner P.J., Gunderson E.P., Konety S.H., Jacobs D.R., Nwabuo C.C., Ebong I.A., Whitham H.K., Goff D.C., Lima J.A. (2016). Association of gestational diabetes mellitus with left ventricular structure and function: The CARDIA study. Diabetes Care.

[40] Lindley K., Williams D., Conner S.N., Verma A., Cahill A.G., Davila-Roman V.G. (2020). The spectrum of pregnancy-associated heart failure phenotypes: An echocardiographic study. Int. J. Cardiovasc. Imaging.

[41] Oliveira A.P., Calderon I., Costa R.A.A., Roscani M.G., Magalhaes C.G., Borges V.T.M. (2015). Assessment of structural cardiac abnormalities and diastolic function in women with gestational diabetes mellitus. Diab. Vasc. Dis. Res..

[42] Gibbone E., Wright A., Vallenas Campos R., Sanchez Sierra A., Nicolaides K.H., Charakida M. (2021). Maternal cardiac function at 19–23 weeks’ gestation in prediction of gestational diabetes mellitus. Ultrasound Obst. Gynecol..

[43] Schannwell C.M., Schneppenheim M., Perings S.M., Zimmermann T., Plehn G., Strauer B.F. (2003). Alterations of left ventricular function in women with insulin-dependent diabetes mellitus during pregnancy. Diabetologia.

[44] Freire C.M.V., Nunes M.C.P., Barbosa M.M., Longo J.R.O., Nogueria A.I., Diniz S.S.A., Machado L.J.C., Oliviera A.R. (2006). Gestational diabetes: A condition of early diastolic abnormalities in young women. J. Am. Soc. Echocardiogr..

[45] Ye X., Li Y., Li Y., Cai Q., Sun L., Zhu W., Ding X., Guo D., Qin Y., Lu X. (2021). Reduced mechanical function of the left atrial predicts adverse outcome in pregnant women with clustering of metabolic risk factors. BMC Cardiovasc. Disord..

[46] Zakovicova E., Charvat J., Mokra D., Svab P., Kvapil M. (2014). The optimal control of blood glucose is associated with normal blood pressure 24 hours profile and prevention of the left ventricular remodeling in the patients with gestational diabetes mellitus. Neuroendocrinol. Lett..

[47] Pintaudi B., Di Vieste G., Corrado F., Creazzo M.F., Valenti A., D’Anna R., Di Benedetto A. (2013). Cardiac diastolic evaluation in pregnant women with abnormal glucose tolerance: An opportunity to detect the early and subclinical alterations and prevent cardiovascular diseases. J. Diabetes Res..

[48] Walker J. (2008). NICE guidance on diabetes in pregnancy: Management of diabetes and its complications from preconception to the postnatal period. Diabet. Med..

[49] Patey O., Carvalho J., Thilaganathan B. (2019). Perinatal changes in fetal cardiac geometry and function in diabetic pregnancy at term. Ultrasound Obstet. Gynecol..

[50] Miranda J., Cerquira R., Ramalho C., Areias J.C., Henriques-Coelho T. (2018). Fetal cardiac function in maternal diabetes: A conventional and speckle-tracking echocardiography study. J. Am. Soc. Echocardiogr..

[51] Aguilera J., Semmler J., Anzoategui S., Zhang H., Nicolaides K.H., Charakida M. (2021). Cardiac function in gestational diabetes mellitus: A longitudinal study from fetal life to infancy. BJOG.

[52] Zablah J., Gruber D., Stoffels G., Cabezas E.G., Hayes D.A. (2017). Subclinical decrease in yocardial function in asymptomatic infants of diabetic mothers: A tissue Doppler study. Pediatr. Cardiol..

[53] Mehta S., Nuamah I., Kalhan S. (1991). Altered diastolic function in asymtomatic infants of mothers with gestational diabetes. Diabetes.

[54] Say L., Chou D., Gemmill A., Tuncalp O., Moller A.B., Daniels J., Gulmezoglu A.M., Temmerman M., Alkema L. (2014). Global causes of maternal death: A WHO systematic analysis. Lancet Glob. Health.

[55] Bellamy L., Casas J., Hingorani A.D., Williams D.J. (2007). Preeclampsia and risk of cardiovascular disease and cencer in later life: Systematic review and metaanalysis. Br. Med. J..

[56] Podymow T., August P. (2010). Postpartum course of gestational hypertension and preeclampsia. Hypertens. Pregnancy.

[57] Wu P., Haththotuwa R., Kwok C.S., Babu A., Kotronias R.A., Rushton C., Zaman A., Fryer A.A., Kadam U., Chew-Graham C.A. (2017). Preeclampsia and future cardiovascular health: A systematic review and meta-analysis. Circ. Cardiovasc. Qual. Outcomes.

[58] Bushnell C., McCullough L.D., Awad I.A., Chireau M.V., Fedder W.N., Furie K.L., Lichtman J.H., Lisabeth L.D., Pina I.L., Reeves M.J. (2014). Guidelines for the prevention of stroke in women: A statement for healthcare professionals from the American heart association/American stroke association. Stroke.

[59] Melchiorre K., Sharma R., Thilaganathan B. (2020). Cardiovascular implications in preeclampsia: An overview. Circulation.

[60] Kraker K., Schutte T., O’Driscoll J., Birukov A., Patey O., Herse F., Muller D.N., Thilaganathan B., Haase N., Dechend R. (2020). Speckle tracking echocardiography:New ways of translational approaches in preeclampsia to detect cardiovascular dysfunction. Int. J. Mol. Sci..

[61] Karlsen S., Dahlslett T., Grenne B., Sjoli B., Smiseth O., Edvardsen T., Brunvald H. (2019). Global longitudinal strain is a more reproducible measure of left ventricular function than ejection fraction regardless of echocardiographic training. Cardiovasc. Ultrasound.

[62] Moors S., van Oostrum N., Rabotti C., Long X., Westerhuis M.E.M., Kemps H.M., Guid Oei S., van Laar J.O.E. (2020). Speckle tracking echocardiography in hypertensive pregnancy disorders: A systematic review. Obstet. Gynecol. Surv..

[63] Clemmensen T., Christiensen M., Logstrup B.B., Kronborg C.J., Knudsen U. (2020). Reduced coronary flow velocity reserve in women with previous preeclampsia: Link to increased cardiovascular disease risk. Ultrasound Obstet. Gynecol..

[64] Reddy M., Wright L., Rolnik D.L., Li W., Mol B.W., La Gerche A., da Silva Costa F., Wallace E.M., Palmer K. (2019). Evaluation of cardiac function in women with a history of preeclampsia: A systematic review and meta-analysis. J. Am. Heart Assoc..

[65] Gibbone E., Wright A., Vallenas Campos R., Sanchez Sierra A., Nicolaides K.H., Charakida M. (2020). Maternal cardiac function at 19–23 weeks’ gestation in prediction of preeclampsia. Ultrasound Obstet. Gynecol..

[66] Vasapollo B., Novelli G., Gagliardi G., Farsetti D., Valensis H. (2020). Pregnancy complications in chronic hypertensive patients are linked to pre-pregnancy maternal cardiac function and structure. Am. J. Obstet. Gynecol..

[67] Youssef L., Miranda J., Paules C., Garcia-Otero L., Vellve K., Kalapotharakos G., Sepulveda-Martinez A., Crovetto F., Gomez O., Gratacos E. (2019). Fetal cardiac remodeling and dysfunction is associated with both preeclampsia and fetal growth restriction. Am. J. Obstet. Gynecol..

[68] Buddeberg B.S., Sharma R., O’Driscoll J.M., Kaelin Agten A., Khalil A., Thilaganathan B. (2018). Cardiac maladaptation in term pregnancies with preeclampsia. Pregnancy Hypertens..

[69] Tadic M., Cuspidi C., Suzic-Lazic J., Vukomanovic V., Mihajlovic S., Savic P., Blagojevic N., Grassi G., Celic V. (2021). Blood-pressure variability is associated with left-ventricular mechanics in patients with gestational hypertension and preeclampsia. Hypertens. Res..

[70] Tadic M., Cuspidi C., Lazic J.S., Vukomanovic V., Mihajlovic S., Savic P., Cvrkotic M., Grassi G., Celic V. (2021). Blood pressure variability correlates with right ventricular strain in women with gestational hypertension and preeclampsia. J. Hum. Hypertens..

[71] De Martelly V., Dreixler J., Tung A., Mueller A., Heimberger S., Fazal A.A., Naseem H., Lang R., Kruse E., Yamat M. (2021). Long-term postpartum cardiac function and its association with preeclampsia. J. Am. Heart Assoc..

[72] Cong J., Fan T., Yang X., Shen J., Cheng G., Zhang Z. (2015). Maternal cardiac remodeling and dysfunction in preeclampsia: A three-dimensional speckle-tracking echocardiography study. Int. J. Cardiovasc. Imaging.

[73] Ersboll A.S., Bojer A.S., Hauge M.G., Johansen M., Damm P., Gustafsson F., Veijlstrup N.G. (2018). Long-term cardiac function after peripartum cardiomyopathy and preeclampsia: A Danish nationwide, clinical follw-up study using maximal exercise testing and cardiac magnetic resonance imaging. J. Am. Heart Assoc..

[74] Clemmensen T.S., Christensen M., Kronborg C.J.S., Knudsen U.B., Løgstrup B.B. (2018). Long-term follow-up of women with early onset pre-eclampsia shows subclinical impairment of the left ventricular function by two-dimensional speckle tracking echocardiography. Pregnancy Hypertens..

[75] Liu W., Li Y., Wang W., Li J., Cong J. (2019). Layer-specific longitudinal strain analysis by speckle tracking echocardiography in women with early and late onset preeclampsia. Pregnancy Hypertens..

[76] Williams D., Stout M.J., Rosenbloom J.I., Olsen M.A., Maddox K.E.J., Deych E., Davila-Roman V.G., Lindley K.J. (2021). Preeclampsia predicts risk of hospitalization for heart failure with preserved ejection fraction. J. Am. Coll. Cardiol..

[77] Zamfirescu M.-B., Ghilencea L.N., Popescu M.-R., Bejan G.C., Ghiordanescu I.M., Popescu A.-C., Myerson S.G., Dorobanțu M. (2021). A practical risk score for prediction of early readmission after a first episode of acute heart failure with preserved ejection fraction. Diagnostics.

[78] Popescu M.R., Panaitescu A.M., Pavel B., Zagrean L., Peltecu G., Zagrean A.M. (2020). Getting an early start in understanding perinatal asphyxia impact on the cardiovascular system. Front. Pediatr..

[79] (2000). National high blood pressure education program working group on high blood pressure in pregnancy. Am. J. Obstet. Gynecol..

[80] Gao Z., Yuan Y., Niu Q.M., Zhang J., Zhao J.J., Guo L.P. (2020). Velocity vector imaging for assessing the heart function in pregnant women with gestational diabetes mellitus. J. Biol. Regul. Homeost. Agents.

[81] Panaitescu A.M., Popescu M.R., Ciobanu A.M., Gica N., Cimpoca-Raptis B.A. (2021). Pregnancy complications can foreshadow future disease—Long-term outcomes of a complicated pregnancy. Medicina.

